# Herbal medicine use and linked suspected adverse drug reactions in a prospective cohort of Ugandan inpatients

**DOI:** 10.1186/s12906-016-1125-x

**Published:** 2016-05-26

**Authors:** Ronald Kiguba, Sam Ononge, Charles Karamagi, Sheila M. Bird

**Affiliations:** Department of Pharmacology and Therapeutics, Makerere University College of Health Sciences, Kampala, Uganda; Department of Obstetrics and Gynaecology, Mulago National Referral Hospital, Kampala, Uganda; Clinical Epidemiology Unit, Makerere University College of Health Sciences, Kampala, Uganda; Medical Research Council Biostatistics Unit, Cambridge, UK

**Keywords:** Adverse drug reactions, Alternative medicines, Herbal medicines, Suspected adverse drug reactions, Uganda

## Abstract

**Background:**

Clinical history-taking can be employed as a standardized approach to elucidate the use of herbal medicines and their linked suspected adverse drug reactions (ADRs) among hospitalized patients. We sought to identify herbal medicines nominated by Ugandan inpatients; compare nomination rates by ward and gender; confirm the herbs’ known pharmacological properties from published literature; and identify ADRs linked to pre-admission use of herbal medicines.

**Methods:**

Prospective cohort of consented adult inpatients designed to assess medication use and ADRs on one gynaecological and three medical wards of 1790-bed Mulago National Referral Hospital. Baseline and follow-up data were obtained on patients’ characteristics, including pre-admission use of herbal medicines.

**Results:**

Fourteen percent (26/191) of females in Gynaecology nominated at least one specific herbal medicine compared with 20 % (114/571) of inpatients on medical wards [20 % (69/343) of females; 20 % (45/228) of males]. Frequent nominations were *Persea americana* (30), Mumbwa/multiple-herb clay rods (23), *Aloe barbadensis* (22), *Beta vulgaris* (12), *Vernonia amygdalina* (11), *Commelina africana* (7), *Bidens pilosa* (7), *Hoslundia opposita* (6), *Mangifera indica* (4), and *Dicliptera laxata* (4). Four inpatients experienced 10 suspected ADRs linked to pre-admission herbal medicine use including *Commelina africana* (4), multiple-herb-mumbwa (1), or unspecified local-herbs (5): three ADR-cases were abortion-related and one kidney-related.

**Conclusions:**

The named herbal medicines and their nomination rates generally differed by specialized ward, probably guided by local folklore knowledge of their use. Clinical elicitation from inpatients can generate valuable safety data on herbal medicine use. However, larger routine studies might increase the utility of our method to assess herbal medicine use and detect herb-linked ADRs. Future studies should take testable samples of ADR-implicated herbal medicines for further analysis.

**Electronic supplementary material:**

The online version of this article (doi:10.1186/s12906-016-1125-x) contains supplementary material, which is available to authorized users.

## Background

Over 60 % of conventional medicines on the global market have been derived either directly or indirectly from natural products, including herbs [1]. Alternative/herbal medicines, hereafter herbal medicines, have a variety of biological properties, among them the ability to contract the uterus and thereby induce abortion and/or reduce post-partum bleeding [2]. In Uganda, as in several other African countries, inducing abortion is illegal unless the physical or mental health of the mother is severely at-risk [3, 4]. Women who seek an abortion may therefore have recourse to the frequently unsafe traditional methods to induce foetal loss [3]. Thus, incomplete abortion or miscarriage is a key reason for admission to gynaecological wards [5]. Outside of Uganda, such admissions have been the focus of previous studies of “unsafe abortion”: for example, in rural Tanzania, where 125 (67 %) of 187 women admitted with incomplete abortion, when interviewed empathetically, revealed that they had experienced unsafe abortion prior to the current admission [6]. Examples of plants used traditionally to procure abortion, whose uterotonic properties have been formally tested and confirmed, include: *Bidens pilosa L., Commelina africana L., Desmodium barbatum (L.) Benth*, *Manihot esculenta Crantz*, *Ocimum suave Klilld., Oldenlandia corymbosa L*., and *Vernonia amygdalina Delile* [3, 7]. The use of different plant parts and solvents to obtain a plant’s extracts might account for differences in the extracts’ biological properties. The highest test concentration of an ethanolic root extract of *Bidens pilosa* (1.40 mg/ml) decreased rat uterine contractility below the level of spontaneous contractions (i.e. was toxic to the uterine muscle) while a similar concentration (1.43 mg/ml) of an aqueous leaf extract of *Bidens pilosa* increased uterine contractions [3, 8].

Other uses of herbs in folk medicine have been reported, some being confirmed by formal testing of the herbs’ pharmacological activity. For instance, *Dicliptera laxata* is used by herbalists in Kenya to treat colorectal cancer [9] and in Uganda as a poison antidote [10], though no published literature was available to confirm these claims. However, *Dicliptera laxata* has antimicrobial [11], anti-inflammatory and antinociceptive properties [12]; traditionally, its roots are chewed as a cough remedy and for stomach pain while its leaf extract is drunk to treat fever, headache, rashes and itching [11, 13].

Extracts of *Bidens pilosa L* are reported to have antihyperglycaemic, antihypertensive, antiulcerogenic, hepatoprotective, antipyretic, immunosuppressive and anti-inflammatory, anti-leukemic, anti-malarial, anti-bacterial, antioxidant, antitumour and antifungal effects [14–17].

*Hoslundia opposita* is used post-partum to cleanse the uterus of any blood clots and heal vaginal lacerations after childbirth. Pharmacological investigations indicate numerous properties of *Hoslundia opposita* including antimicrobial, analgesic, anti-inflammatory, antimalarial, antidiabetic, acaricidal and central nervous system activity [18–22].

Our research-team’s approach was only as empathetic as clinical history-taking at its best but had the advantage of being standardized: the same questions about herbal medicine use in the 4-weeks prior to hospital admission were posed to representatively sampled patients in three medical wards and one gynaecological ward. Moreover, adverse event reporting for herbal medicines [23] employed the existing routine clinical approach to elucidate the safety of herbs without the need for sophisticated and costly methods, or the use of the not well-studied ethnopharmacovigilance concept [24]. Our study aimed to identify the herbal medicines nominated by Ugandan inpatients; compare the nomination rates by ward and gender; confirm the herbs’ known pharmacological properties from published literature; and identify suspected adverse drug reactions (ADRs) linked to pre-admission use of herbal medicines.

## Methods

### Study design and setting

The study site was the 1790-bed Mulago National Referral Hospital [25] which receives an annual inpatient turnover of more than 140,000 patients. The study setting, design and data collection/management have been described elsewhere [26]. The study setting comprised three medical wards [Infectious Diseases and Gastrointestinal Illnesses (IDGI); Haematology, Neurology and Endocrinology (HNE); Cardiovascular, Pulmonology and Nephrology (CPN)] and one Gynaecological ward (GYN). Each of the four wards has an official bed capacity of 54 but can receive 70 to 80. Admissions on the medical wards average 10–15 patients per day in each of wards IDGI & CPN and 5–10 patients per day in HNE, thus about 25–40 medical wards admissions per day; and 20–25 admissions per day on the GYN ward.

### Data collection

During October to November 2013, a pilot phase was conducted on all four wards to assess the feasibility of undertaking the cohort study and to refine study instruments. The pilot data, however, are excluded from the final analyses. The main study commenced in December 2013 to April 2014 when research teams recruited patients on the study wards according to a systematic random sampling procedure with three new admissions per day on long-stay wards (HNE/CPN) and six per day on short-stay wards (IDGI/GYN). Each ward-team purposed to select at random one of the first two (IDGI), three (HNE), and four (CPN/GYN) new admissions, and subsequently every second, third, and fourth admission respectively. Voluntary participation of the inpatients (≥18 years) was sought through provision of written informed consent.

On Day 1 of recruitment, we assessed medication use and ADRs among the inpatients. Each ward’s research team interviewed the patients and/or their caregivers to obtain relevant baseline data on demographics, clinical conditions, and medications including herbal medicines; and subsequently conducted daily assessments until discharge, transfer, death, or loss to follow-up. The elicited information included details on the use of herbal medicines in the 4-weeks prior to hospitalization and, where possible, the specific names of these herbal remedies.

Patients who were coded as having used herbal medicines in the 4-weeks prior to hospital admission but for whom no herbal medicine was listed were counted as **not** having taken a *nominated herbal medicine*. We provide sub-totals for these exclusions. Patients were counted as having taken a nominated herbal medicine if they answered “no” to the lead-in question about having taken herbal medicines in the previous 4-weeks, yet they nominated herbal remedies they had taken at admission or in the previous 4-weeks on a subsequent form where questions on herbal medicines used were posed as part of routine clinical elicitation of all medicines. For completeness, patients’ responses were counted as “yes” even if their nomination was as vague as “unknown liquid”.

First, we identified the herbal medicines nominated by the women admitted to our study from the GYN ward at Mulago Hospital. Nominations of herbal medicines were then compared between the women admitted for abortion-related reasons and: i) the recruited females admitted to GYN for other reasons; ii) the females admitted to the medical wards; and iii) the males recruited from the medical wards. Next, we identified the Latin botanical names for plant remedies which were frequently nominated by patients in our GYN ward or differentially nominated between patients in GYN and the three medical wards (using a minimum of at least four nominations as our criterion for selection). For the selected plants with linked Latin botanical name, we sought confirmation from the published literature about their known pharmacological properties. Lastly, we identified the suspected ADRs linked to the pre-admission use of herbal medicines. A *suspected ADR* was any undesirable medical occurrence that developed after the administration of medicine and for which there was, at least, a **possible** causal relationship between the medicine and the event as measured by the Naranjo ADR Probability Scale [27, 28]. Seriousness was assessed using the WHO Uppsala Monitoring Centre (UMC) criteria [29]. Consensus agreement on ADR causality and seriousness was done in a committee headed by the ward-based study physician and senior clinical pharmacist (principal author) [26].

### Data analysis

Descriptive statistics on nomination-frequency are presented separately for the Gynaecological ward, female inpatients in the three medical wards, and male inpatients in the three medical wards; and for the inpatients in the GYN ward according to whether (or not) their admission was abortion-related. Posthoc comparison of nomination rates by ward, gender and specific herbal medicine was done using *χ*^2^ tests to assess statistical differences.

For the more frequently-nominated plant remedies for which the Latin botanical name was ascertained and biological properties other than abortifacient were identified, the clinical history of the associated patients – given by working diagnoses - was summarized to discover if any of the patients’ diagnoses or co-morbidities aligned with the plant’s identified biological properties.

## Results

Table 1 summarizes, by ward and gender, the available information on herbal medicine use by 176 (79 %) of 222 inpatients who nominated at least one specific or vague herbal medicine. One in seven of the female inpatients in GYN (14 %, 26/191) nominated at least one specific herbal remedy, as detailed in Table 2. By comparison, specific nominations were made by 19 % (39/201) of females and 24 % (29/119) of males in the IDGI ward; by 31 % (22/72) of females and 18 % (8/45) of males in HNE; and by 11 % (8/70) of females and 12 % (8/64) of males in CPN. The proportion of inpatients on CPN (37 %, 16/43) that specified the herbal medicine used was only half that in the other two medical wards: IDGI at 73 % (68/93) and HNE at 75 % (30/40). The specification rate of herbal medicines on CPN was also lower than in the GYN which achieved specific nominations by 57 % (26/46) of patients who gave some indication of having used herbal medicines in the 4-weeks prior to admission. In summary, across the three medical wards, specific nominations of at least one herbal remedy used in the 4-weeks prior to, or at, admission were made by 20 % (69/343) of females and by 20 % (45/228) of males.

**Table 1:** Patients who nominated specific herbal medicines that they had used in the 4-weeks prior to hospital admission: by ward and gender, Uganda, 2014

Herbal medicine use	Ward and gender						
	GYN: Gynaecology, *n*	IDGI: Infectious Diseases & Gastrointestinal Illnesses, *n*		HNE: Haematology, Neurology & Endocrinology, *n*		CPN: Cardiovascular, Pulmonology & Nephrology, *n*	
	Abortion-related + Other admissions	Female	Male	Female	Male	Female	Male
No declared use in the past four weeks	61 + 84 = 145	145	82	44	33	46	45
Coded yes, but no details	7 + 5 = 12	8	5	6	1	11	3
Only vague nominations of remedies used	5 + 3 = 8	9	3	0	3	5	8
At least one specific remedy nominated	11 + 15 = 26	39	29	22	8	8	8
Totals	107 + 84 = 191	201	119	72	45	70	64

**Table 2:** Specific herbal medicines nominated by 176 out of 762 hospitalized patients at Mulago Hospital, Uganda, 2014

Traditional herbal medicine		No. of citations					Commentary
Local name	Botanical name	GYN (*N* = 191)		Medical Wards IDGI, HNE & CPN (*N* = 571)		Total	
		Abortion-related admissions (*n* = 107)	Other admissions (*n* = 84)	Female (*n* = 343)	Male (*n* = 228)		
Avocado leaves	*Persea americana*	1	1	17	11	30	Local knowledge: Used mainly in patients with anaemia to raise their haemoglobin levels
Mumbwa	–	6	7	5	5	23	Local knowledge: multiple herb-containing clay rods administered orally. Assumed to serve as wellbeing supplement for the foetus and mother.
Kigagi (Aloe vera)	*Aloe barbadensis*	0	0	12	10	22	Treats fever
Beet root	*Beta vulgaris*	0	0	7	5	12	For treatment of anaemia
Mululuza	*Vernonia amygdalina*	0	0	6	5	11	Local knowledge: Treats fever, malaria
Nanda	*Commelina africana*	2	2	1	2	7	Local knowledge: Induces abortion. Inserted adjacent to the cervix to induce it to dilate. Literature: Hypoglycaemic properties
Sere (Blackjack)	*Bidens pilosa*	1	0	4	2	7	Wide range of biological properties including significant antibacterial and antifungal activity
Kamunye	*Hoslundia opposita*	3	1	1	1	6	Locally: Cleanses uterus, treats vaginal lacerations after childbirth and fever. Literature: Anti-inflammatory and wound healing
Mango leaves/mangoes	*Mangifera indica*	0	0	4	0	4	For anaemia
Muzukizi	*Dicliptera laxata*	0	0	2	2	4	Local knowledge: Treats colorectal cancer, poison antidote
Kiyondo	*Kalanchoe pinnata Lam*	1	0	0	0	1	None of these three herbs was mentioned by any of the inpatients on the IDGI, HNE and CPN wards. Local knowledge: Kiyondo is aphrodisiac Literature: Kiyondo - anticonvulsant, antidiabetic and wound healing. Gwalimu - antibacterial and anti-inflammatory.
Gwalimu	*Maytenus senegalensis* (Lam) Exell	1	0	0	0	1	
Ekigaranga		1	0	0	0	1	

Table 2 documents the frequency of specific nominations by inpatients on the study wards, provides Latin botanical names, and for the most frequently nominated, provides commentary on their pharmacological activity and local folklore knowledge. On the GYN ward, Mumbwa (clay rod) received 13 citations followed by Nanda (*Commelina africana*) and Kamunye (*Hoslundia opposita*) which received four mentions each, see also Additional file 1: Table S1. On the three medical wards (IDGI, HNE, CPN), frequent nominations were of avocado leaves (28), aloe vera (22), beet root (12), mululuza (11), and mumbwa (10). Twenty-six (31 %) of the 83 female inpatients on the medical wards who made specific or vague nominations reported at least two nominations, as did 20 % (12/59) of male-nominators.

Mululuza (*Vernonia amygdalina*) had 11 mentions overall, none from the GYN ward. Furthermore, Nanda (*Commelina africana*) and Sere (*Bidens pilosa*) each had seven mentions, Kamunye (*Hoslundia opposita*) had six mentions and Muzukizi (*Dicliptere Laxata*) had four mentions, see Table 2. Just below this threshold was Kazire (proprietary herbal concoction which contains mainly aloe vera) with three mentions, see Additional file 1: Table S2.

Nanda’s nominations by seven inpatients included two males from CPN whose working diagnoses were: i) newly diagnosed with immunosupressed syndrome (ISS) and acute gastroenteritis, oral candidiasis and lymphoma; and ii) community-acquired pneumonia with pulmonary tuberculosis.

Kamunye’s six nominations included two patients in CPN, one of them male, whose clinical histories were: i) male with pulmonary tuberculosis and ISS; and ii) female with hypertensive heart disease in failure, and severe hypertension.

Mululuza was not cited by any of the 191 female patients on GYN (versus 2.8 expected) but 11 mentions were made by 571 patients in medical wards (versus 8.2 expected), a marginally statistically significant posthoc comparison. Eight of Mululuza’s 11 mentions were from the 320 inpatients in IDGI.

Sere (*Biden pilosa*) was mentioned in all four wards and by both sexes (five females; two males), see Additional file 1: Table S3 for their diverse clinical histories and for those of four patients who nominated Musukizi (*Dicliptera laxata*).

Three herbal remedies were each nominated once by the 107 abortion-related patients in GYN but not once by any of the 343 females or by any of the 228 male patients on the medical wards. This may be happenstance. Nonetheless, we tried to establish the botanical names for Ekigaranga, Gwalimu (also called Naligwalimu), and Kiyondo (also called Ekiyondo) in order to investigate their published properties (if any); and were successful for Kiyondo (*Kalanchoe pinnata Lam*), which has anticonvulsant, antidiabetic and wound healing properties and Gwalimu [*Maytenus senegalensis* (Lam) Exell] with antibacterial and anti-inflammatory activity, see Table 2 & Additional file 1: Table S1.

Finally, Table 3 shows the suspected ADRs linked to the pre-admission use of herbal medicines: 10 suspected ADRs were experienced by four patients, three of whom were from GYN and one from CPN. Except for vaginally inserted Nanda (*Commelina africana*) stick, all the other ADR-implicated herbal remedies were administered orally.

**Table 3:** Suspected ADRs linked to the use of herbal medicines in the 4-weeks prior to hospital admission

Pt No.	Herbal medicine	Suspected ADR	Serious	Causality	Ward	Working diagnosis
Experienced herbal medicine-related suspected ADRs only						
1.	Mumbwa	Abdominal pain with associated contractions	Yes	Probable	GYN	Complete abortion, Urinary tract infection, Inevitable abortion
2.a)	Nanda stick	Vaginal bleeding	No	Possible	GYN	Septic abortion
		Dysuria	Yes	Possible		
2.b)	Local herbs	Diarrhoea	Yes	Possible		
		Lower abdominal pain	Yes	Possible		
Experienced other non-herbal medicine-related suspected ADRs						
3.	Nanda	Bloody vomitus	Yes	Possible	GYN	Missed abortion, gastrointestinal infection
		Bloody diarrhoea	Yes	Possible		
4.	Unknown liquid	Hyponatremia	Yes	Possible	CPN	Acute kidney injury secondary to toxins from herb, glomerular nephritis, severe anaemia
		Abdominal distension	No	Possible		
		Anaemia^a^	Yes	Possible		

Source of herbal medicines: patient no. 1 - Auntie; patient no. 2 - sister, patient no. 3 - self-medication, and patient no. 4 - traditional herbalist. Except for vaginally inserted nanda stick, all other herbal remedies were taken orally

^a^ADR causation was also linked to ceftriaxone and/or captopril use

## Discussion

### Frequency of nominated herbal medicines by ward and gender

The specific herbal medicines nominated by inpatients on the various specialized wards generally differed, probably guided by local folklore knowledge of their use. Mumbwa, which are clay rods constituted with one or more local herbs, received the highest number of citations on the Gynaecological ward (GYN); however, patients were seldom aware of the specific herbs that were compounded in these clay rods. Nanda (*Commelina africana*) was the next most frequently cited herb on GYN and is known to contract the uterus [7]. *Commelina africana* is normally administered orally; however, it was inserted vaginally as a stick by one woman admitted on GYN who poked her cervix/uterus to induce abortion. Kamunye (*Hoslundia opposita*) was another commonly nominated herb which, according to local obstetricians, is traditionally used post-partum to cleanse the uterus of any blood clots and heal the vaginal lacerations caused by childbirth. *Hoslundia opposita* is reported in the literature to have anti-inflammatory (linked to saponin-content) and wound healing (linked to coumarin-content) properties [30, 31], which is consistent with *Hoslundia opposita*’s local use for gynaecological conditions, as mentioned above. Sere (*Bidens pilosa*), was used on all four wards and by both males and females. Sere is reported, in folk medicine, to treat various disease conditions, which is attributed to its varied biological activity including antibacterial, antifungal, anti-inflammatory, antipyretic, antimalarial, antihyperglycaemic, antihypertensive, hepatoprotective, antiulcerogenic, and anticancer properties, among others [14–16].

The specific nomination rate of herbal medicines was lowest on the CPN ward when compared with the other wards; however there was no difference in nomination rate by gender. Mumbwa received a noticeable and comparable number of citations on the three medical wards vs. the GYN ward. However, avocado leaves (*Persea americana*), aloe vera (*Aloe barbadensis*), beet root (*Beta vulgaris*), mululuza (*Vernonia amygdalina*), and muzukizi (*Dicliptera laxata*) were predominantly cited on the medical wards. Avocado leaves and beet root are used for anaemia. *Commelina africana* was mentioned with similar frequencies on both the medical and gynaecological wards. In addition to its uterotonic properties, *Commelina africana* is reported to have hypoglycaemic effects [32]. *Vernonia amygdalina* is used traditionally to treat malaria: and the herb’s antimalarial activity has been validated [33]. *Dicliptera laxata* is used by herbalists in Kenya to treat colorectal cancer [9] and as antidote to poison in Uganda [10]: however, studies to document the anticancer and poison antidote properties of this herb are scarce. *Dicliptera laxata* is reported to have antimicrobial, anti-inflammatory and antinociceptive properties, with its leaves being used to treat rashes and itching [11, 12]: the herb is also used to treat headache in Ethiopia [13].

Among the three herbs nominated on the GYN ward but not on the three medical wards, Kiyondo (*Kalanchoe pinnata Lam*) in local Ugandan folklore knowledge is used to dry a newborn’s umbilical cord [34], treat morning sickness in pregnant women and also has aphrodisiac properties. *Kalanchoe pinnata Lam* is proven to have anticonvulsant, antidiabetic and wound healing properties [35–37]. Gwalimu [*Maytenus senegalensis* (Lam.) Excell] is used locally to treat infections and inflammatory conditions; and has been demonstrated to have antibacterial and anti-inflammatory properties [38]. Gwalimu is also used traditionally to treat fertility-related problems. Published information and local folklore knowledge on Ekigaranga was scarce.

We focused on the GYN herbals to identify “new” abortifacients but this did not succeed. However, we highlighted known abortifacients. We also compared GYN versus the medical wards nomination rates (and also by gender) to identify herbals with other active principles, which worked insofar as those identified are already known. The methodological approach used, we believe, was sound although we clearly needed more than 762 cases to make “discoveries”. Furthermore, patients ought to specify the herbal remedies they have taken, which they usually consume to complement conventional western medication. Out of fear, though, the patients may deliberately conceal the required information on herbal medicine use [39]. Thus, the inquiry skills of the clinical assessment teams should improve, especially on the CPN ward, to increase the chances of eliciting, from the patients, specific information on herbal medicine use.

A considerable proportion of women on GYN (56 %, 107/191) presented with abortion-related diagnoses, which is comparable with the proportion of Tanzanian women (67 %, 125/187) with incomplete abortions who conceded having had unsafe abortion prior to their current hospitalization [6]. Half the Tanzanian women had resorted to traditional providers and, in these cases, plant species were often used as abortion remedies. However, only one in five Ugandan inpatients with abortion-related diagnoses reported having used herbal medicines in the 4-weeks prior to hospital admission. The annual global estimate of unsafe abortions in women of reproductive age (15–44 years) is 14 per 1000 women and is higher in sub-Saharan Africa (31 per 1000) than in Eastern Europe (6 per 1000) [40]. In addition, the abortion-attributable proportion of maternal mortality is higher in Eastern Africa (18 %) than in Eastern Europe (11 %), probably because abortion is illegal in Eastern Africa [41].

### Community-acquired suspected ADRs linked to pre-admission use of herbal medicines

No serious suspected ADR was linked definitively to herbal medicine use. Three of the four suspected ADRs that implicated herbal medicine use occurred in patients with abortion-related working diagnoses, in two of whom the intention to abort was explicit. However, the one case of acute kidney injury linked to herbal medicine use in the community might **not** have been deliberate. Unsafe use of herbal medicines to induce abortions can lead to a high burden of overt preventable ADRs: this information may be relatively easier to elicit clinically from patients. However, it is the more subtle preventable ADRs from herbs when used for other therapeutic purposes which, in the absence of appropriate laboratory support and expertise in clinical diagnosis, might be difficult to link to herbal medicine use and could thus go unnoticed but silently continue to harm patients.

Four in five patients who reported use of herbal medicines during the 4-weeks prior to admission nominated at least one specific or vague herbal medicine during routine clinical elicitation of all medicines. Clinical elicitation is therefore a powerful tool for clinicians to obtain, from patients, useful medication history of both herbal and conventional western medicines to better conduct medicines reconciliation and subsequently provide appropriate pharmaceutical care to inpatients. Concurrent use of conventional and herbal medicines, more so in the community where there is little supervision, may promote herb-drug interactions and increase the risk of ADRs [42]. Overall, four patients (2 %, 4/222), or one in 50, experienced at least one community-acquired suspected ADR linked to pre-admission use of a herbal remedy signalling a relatively frequent occurrence of patient-reported suspected ADRs linked to pre-admission herbal remedies. Excluding abortion-related suspected ADRs, only one patient in 222 (0.5 %) who coded yes for the use of herbal remedies might have experienced an unintentional community-acquired suspected ADR. Further investigation of the latter rate of 5 per 1000 patients requires a much larger, preferably routine PV system with integrated patient-reporting, and/or pharmacoepidemiological studies to track and assess the safety of herbal remedies used by patients. The patient-reported suspected ADR estimates, however, cannot be generalized to the wider community since the herb-linked suspected ADRs themselves and/or failure by the patients to have sought conventional treatment may have provoked admission. However, a herb-linked ADR estimate of ~5 per 1000 inpatients is sufficiently high for ward staff to pay heed to. We did not take from patients specimens of the consumed herbal medicines and, in some instances, not even the herb constituents could be inferred, which is a limitation. Hospital clinicians need routinely to ask about and periodically obtain, for formal testing, samples of herbal remedies taken by the one in 50 patients with a herb-linked suspected ADR, which might increase the ability to identify definite ADRs. Caution is advised as the low occurrence rate of reported unintentional herbal medicine-linked ADRs may be due to non-disclosure by the patients [39]. That limitation notwithstanding, our systems need to streamline ADR reporting for conventional medicines before the safety monitoring of herbal medicines can become a genuine reality.

Using logistic regression, our team previously constructed risk-scores for developing a suspected hospital-acquired ADR for which one of the key covariates was pre-admission use of herbal medicines. Those who had used herbal medicines during the 4-weeks prior hospital admission rarely specified the herbs they had taken [43]. A possible explanation for the failure by patients to specify their herbal medicines may be unlabelled herbal formulations with concoctions/mixtures of several different herbs that patients could not themselves identify [39]. If so, might the multiple herbs per concoction/mixture, when taken by the patient, have led to a higher likelihood of herb-drug interactions in the hospitalized patients? Future surveillance should ensure that specimens of the herbs taken by patients with linked suspected ADRs are obtained and tested.

## Conclusions

The named herbal medicines and their nomination rates generally differed by specialized ward, probably guided by local folklore knowledge of their use. Clinical elicitation from inpatients can generate valuable safety data on herbal medicine use. However, larger routine studies might increase the utility of our method to assess herbal medicine use and detect herb-linked ADRs. Future studies should take testable samples of ADR-implicated herbal medicines for further analysis.

## Additional file

Additional file 1: Table S1. Specific herbal medicines nominated by 34 female patients in the Gynaecological ward, Uganda, 2014. **Table S2.** Specific herbal medicines nominated by 142 female and male patients in the Medical wards, Uganda, 2014. **Table S3.** Clinical histories of inpatients who nominated Sere (*Bidens pilosa*) and Muzukizi (*Dicliptera laxata*), Uganda, 2014. (DOCX 26 kb)

## Abbreviations

ADRs: adverse drug reactions
CPN: Cardiovascular, Pulmonology and Nephrology
GSK: GlaxoSmithKline
GYN: Gynaecology
HNE: Haematology, Neurology and Endocrinology
IDGI: Infectious Diseases and Gastrointestinal Illnesses
WHO: World Health Organization
