# Supplementary material for: Herbal medicine use and linked suspected adverse drug reactions in a prospective cohort of Ugandan inpatients
Source: BMC Complement Altern Med. 2016 May 26;16:145. doi: 10.1186/s12906-016-1125-x (PMC4881043; doi:10.1186/s12906-016-1125-x)
Supplement: Additional file 1: Table S1. — Specific herbal medicines nominated by 34 female patients in the Gynaecological ward, Uganda, 2014. Table S2. Specific herbal medicines nominated by 142 female and male patients in the Medical wards, Uganda, 2014. Table S3. Clinical histories of inpatients who nominated Sere (Bidens pilosa) and Muzukizi (Dicliptera laxata), Uganda, 2014. (DOCX 26 kb) [file 12906_2016_1125_MOESM1_ESM.docx]

| **Table S1. Specific herbal medicines nominated by 34 female patients in the Gynaecological ward, Uganda, 2014** | | | | | | |
| --- | --- | --- | --- | --- | --- | --- |
| **Plant remedy** | | **107 Abortion-related Admissions to GYN** | **84 Other Admissions to GYN** | | **Commentary** | |
|  | |  |  | |  | |
| **Specific alternative/herbal medicines** | | | | | | |
| Mumbwa or Emumbwa | | 6 | 7 | | Local knowledge: multiple herb-containing clay rods administered orally. Assumed to serve as wellbeing supplement for the foetus and mother. | |
| Nanda | | 2 | 2 | | *Commelina africana*  Local knowledge: Induces abortion. Inserted adjacent to the cervix to induce it to dilate.  Literature: Hypoglycaemic properties | |
| Kamunye | | 3 | 1** | | *Hoslundia opposita*  Local knowledge: Cleanses the uterus and treats vaginal lacerations after childbirth. It also treats fever.  Literature: Has anti-inflammatory and wound healing properties | |
| Avocado leaves | | 1* | 1 | | *Persea americana*  Local knowledge: Used mainly in patients with anaemia to raise their haemoglobin levels | |
| Avocado seed | |  | 1** | |  |  |
| Ekigaranga | | 1 |  | | None of these three herbs was mentioned by any of the 343 female patients on wards IDGI, HNE and CPN, or by any of the 228 male patients. Local knowledge: Kiyondo is used as an aphrodisiac. Literature: Kiyondo - anticonvulsant, antidiabetic and wound healing. Gwalimu - antibacterial and anti-inflammatory activity | |
| Gwalimu | | 1 |  | |  |  |
| Kiyondo | | 1 |  | |  |  |
| Sere (Blackjack) | | 1* |  | | *Bidens pilosa*  Wide range of biological properties including significant antibacterial and antifungal activity | |
|  | |  |  | |  | |
| **Vague nominations** | | | | | | |
| Herbal | | 1 | 1 | |  | |
| Liquid for appetite | | 1 |  | |  | |
| Liquid for treating ulcers | | 1 |  | |  | |
| Liquid (unknown) | |  | 1 | |  | |
| Liquid – herbal | |  | 1 | |  | |
| Local herb | | 1, cited additionally by one of the nanda patients above |  | |  | |
| Local liquid (unknown) | |  | 1 | |  | |
| *Cited by the same patient; **Cited by the same patient | | | | | | |
| **Table S2. Specific herbal medicines nominated by 142 female and male patients in the Medical wards, Uganda, 2014** | | | | | | |
| **Plant remedy** | **Female Admissions to IDGI, HNE & CPN wards made specific or vague nominations of herbal remedies (83/343)** | | | **Male Admissions to IDGI, HNE & CPN wards made specific or vague nominations of herbal remedies (59/228)** | | **Commentary** |
|  | | | | | | |
| **Specific herbal medicines** | | | | | | |
| Mumbwa or Emumba | 1^3^1111 | | | 11^4^1^5^11 | | Local knowledge: multiple herb-containing clay rods administered orally. Assumed to serve as wellbeing supplement for the foetus and mother. |
| Nanda | 1^22^ | | | 11^10^ | | *Commelina africana*  Local knowledge: Induces abortion. Inserted adjacent to the cervix to induce it to dilate.  Literature: Hypoglycaemic properties |
| Kamunye | 1 | | | 1 | | *Hoslundia opposita*  Local knowledge: Treats fever and stomach wounds in HIV/AIDS patients. Also cleanses the uterus and treats vaginal lacerations after childbirth.  Literature: Has anti-inflammatory and wound healing properties |
| Akasaala | 1 | | |  | |  |
| Aloe vera | 11^1^111111^9^1^12^11 | | | 1^2^1111^6^1^8^1^11^ | | *Aloe barbadensis*  Treats fever |
| Avocado leaves | 1^1^1^2^1^7^1^9^11^11^11^14^111^15^  11^19^11^21^1^23^1^24^ | | | 111^2^11^3^1^5^1111^7^1^9^ | | *Persea americana*  Local knowledge: Used mainly in patients with anaemia to raise their haemoglobin levels |
| Balaalo herbal mixture | 1 | | |  | |  |
| Bamasiko herb |  | | | 1^4^ | |  |
| Bamutekaga mixture | 1 | | |  | |  |
| Bazira cough mixture | 1 | | |  | |  |
| Beet root (juice) | 1111^13^11^19^1^21^ | | | 11111 | | *Beta vulgaris*  For treatment of anaemia |
| Brother Anatoli (mixture) | 1^5^1^18^ | | |  | |  |
| Bukedde Butya | 1 | | |  | |  |
| Buyinza | 1^22^ | | |  | |  |
| Buzira | 1 | | |  | |  |
| Dongolo soap | 1^12^ | | |  | |  |
| Ebbombo or Akabombo | 11^26^ | | |  | | *Cyphostemma adenacuale*  Induces uterine contractions  *Momordica Foetida*  Used locally to treat cough |
| Ejirikiti | 1^4^1^16^ | | |  | | Erythrina abyssinica  Local knowledge: Treats fever, weakness, cough. |
| Ekikatula herbal drink |  | | | 1 | |  |
| Ekisanda |  | | | 1 | |  |
| Enkolimbo |  | | | 1^1^ | |  |
| Enzirugaze bark | 1^17^ | | |  | |  |
| Fenuplus | 1 | | |  | |  |
| Forever Epiblanc, topical | 1 | | |  | |  |
| Garlic (mixture) | 1^5^ | | |  | | *Allium sativum* |
| Garlic+onion juice | 1^6^1^14^ | | |  | |  |
| Herbal drink from stem of mango tree |  | | | 1 | |  |
| Hibiscus rosera/Kerekedde | 1^1^ | | | 1 | |  |
| Honey |  | | | 1 | |  |
| Kabuti |  | | | 1 | |  |
| Kakata | 1^8^ | | |  | |  |
| Kamubiri | 1 | | |  | |  |
| Kazire, proprietary name | 1 | | | 1^7^1^12^ | |  |
| Kibwakulata |  | | | 1 | |  |
| Kifumufumu | 1 | | |  | |  |
| Kigagi (mixture) – otherwise known as aloe vera | 1^20^ | | | 111^12^ | |  |
| Kisakyamuzadde | 1^22^ | | |  | |  |
| Lucas cough mixture | 1 | | |  | |  |
| Mango leaves/mangoes | 1^2^1^15^1^24^1^26^ | | |  | |  |
| Matovu | 1^17^ | | | 1^1^ | |  |
| Matugunda | 1^1^ | | |  | |  |
| Mukenkerere Mixture | 1^3^ | | |  | |  |
| Mululuza or omululuza) | 11^6^1^8^1^9^11^20^ | | | 11^2^1^4^1^6^1^8^ | | *Vernonia Amygdalina*  Local knowledge: Treats fever, malaria  Literature: antimalarial activity |
| Mululuza/guava mixture | 1 | | |  | | *Psidium Guajava* |
| Munyanya | 1^1^ | | |  | |  |
| Muzukizi | 1^13^1^23^ | | | 11^7^ | | *Dicliptere Laxata*  Locally used to treat colorectal cancer, poison antidote |
| Mussa | 1 | | |  | | *Kigelia africana* |
| Mutuba root | 1^7^ | | |  | |  |
| Namirembe herbal drink |  | | | 1 | |  |
| Ngetwa herbal medicine | 1 | | |  | |  |
| Nkolimbo | 1^16^ | | |  | |  |
| Omuwafu | 1^4^ | | |  | | *Canarium schweinfurthii* |
| Paw Paw leaves |  | | | 1^3^ | |  |
| Rashid Lukwago herbal mixture | 1 | | |  | |  |
| Sere (Blackjack) | 1^11^11^17^1^18^ | | | 1^1^1^9^ | | *Bidens pilosa*  Wide range of biological properties including significant antibacterial and antifungal activity |
| Spinach & green pepper | 1^14^ | | |  | |  |
| Suubi drink |  | | | 1 | |  |
| Top plus |  | | | 1^11^ | |  |
| Vicks kingo | 1 | | | 1 | |  |
|  | | | | | | |
| **Vague nominations** | | | | | | |
| Chinese herbal tablet | 1^10^ | | |  | |  |
| Concoction for diabetes |  | | | 1 | |  |
| Cough mixture | 1 | | |  | |  |
| Herbal | 111111^10^1^13^ | | | 11 | |  |
| Herbal smear for legs | 1 | | |  | |  |
| Herbal medicine for chest pain |  | | | 1 | |  |
| Herbal liquid for cough |  | | | 1 | |  |
| Liquid for treating ulcers |  | | |  | |  |
| Liquid (unknown) for hypertension | 1 | | |  | |  |
| Liquid (unknown) | 11 | | | 1^10^1111 | |  |
| Liquid – herbal |  | | | 1111 | |  |
| Local herb | 1 | | |  | |  |
| Miscellaneous medicines |  | | | 1 | |  |
| Powder mixed with vaseline | 1^12^ | | |  | |  |
| Topical herbal smear | 1^25^ | | |  | |  |
| Topical herbs for chest pain | 1^25^ | | |  | |  |
| Ulcer mixture | 1 | | |  | |  |
| Separately for each gender, numerical superscripts link remedies nominated by the same patient. Nominations by HNE ward patients are highlighted in turquoise, by CPN ward patients in yellow. | | | | | | |

| **Table S3: Clinical histories of inpatients who nominated Sere (*Bidens pilosa*) and Muzukizi (*Dicliptera laxata*), Uganda, 2014** | | | |
| --- | --- | --- | --- |
| **Herbal remedy** | **Gender** | **Ward** | **Working diagnoses** |
| Sere (*Bidens pilosa*) | Male | CPN | Right pleural effusion secondary to pulmonary tuberculosis and pneumonia |
|  | Male | IDGI | Acute cholecystitis, Speticaemia |
|  | Female | GYN | Incomplete abortion and moderate anaemia |
|  | Female | HNE | Immunosuppressed syndrome (ISS), Deep vein thrombosis |
|  | Female | HNE | HHS, Hypertension, Diabetes type II, pyelonephritis, urosepsis, hypotension, pneumonia |
|  | Female | HNE | Severe anaemia |
|  | Female | IDGI | Newly diagnosed hypertension and no chronic illnesses prior to admission for septicaemia, malaria and electrolyte imabalance. |
| Muzukizi (*Dicliptera laxata*) | Female | HNE | Zidovudine-induced anaemia, hypotension |
|  | Female | CPN | Pleural effusion, disseminated tuberculosis, peptic ulcer disease, extrapulmonary tuberculosis, pulmonary tuberculosis |
|  | Male | IDGI | Upper gastrointestinal bleeding, malaria, urinary tract infection, malignancy, peptic ulcer disease |
|  | Male | HNE | Sickle cell disease with vasocclusive crisis, bronchopneumonia, asthma, sepsis, left tibia osteomyelitis |
